# Synergism of the receptor tyrosine kinase Axl with ErbB receptors mediates resistance to regorafenib in hepatocellular carcinoma

**DOI:** 10.3389/fonc.2023.1238883

**Published:** 2023-09-08

**Authors:** Kristina Breitenecker, Viola Hedrich, Franziska Pupp, Doris Chen, Eva Řezníčková, Gregor Ortmayr, Heidemarie Huber, Gerhard Weber, Lorenz Balcar, Matthias Pinter, Wolfgang Mikulits

**Affiliations:** ^1^ Center for Cancer Research, Comprehensive Cancer Center, Medical University of Vienna, Vienna, Austria; ^2^ Department of Chromosome Biology, Max Perutz Labs Vienna, University of Vienna, Vienna, Austria; ^3^ Department of Experimental Biology, Faculty of Science, Palacký University Olomouc, Olomouc, Czechia; ^4^ Department of Internal Medicine III, Division of Gastroenterology & Hepatology, Medical University of Vienna, Vienna, Austria

**Keywords:** hepatocellular carcinoma, GAS6, Axl, tyrosine kinase inhibitors, Regorafenib, ErbB, bFGF

## Abstract

**Introduction:**

Hepatocellular carcinoma (HCC) patients at advanced stages receive immunotherapy or treatment with tyrosine kinase inhibitors (TKIs) such as Sorafenib (Sora) or Lenvatinib in frontline as well as Regorafenib (Rego) or Cabozantinib in second-line. A major hindrance of TKI therapies is the development of resistance, which renders drug treatment futile and results in HCC progression.

**Methods:**

In this study, we addressed the impact of the receptor tyrosine kinase Axl binding to its ligand Gas6 in acquiring refractoriness to TKIs. The initial responses of Axl-positive and Axl-negative cell lines to different TKIs were assessed. Upon inducing resistance, RNA-Seq, gain- and loss-of-function studies were applied to understand and intervene with the molecular basis of refractoriness. Secretome analysis was performed to identify potential biomarkers of resistance.

**Results:**

We show that HCC cells exhibiting a mesenchymal-like phenotype were less sensitive to drug treatment, linking TKI resistance to changes in epithelial plasticity. Gas6/Axl expression and activation were upregulated in Rego-resistant HCC cells together with the induction of ErbB receptors, whereas HCC cells lacking Axl failed to stimulate ErbBs. Treatment of Rego-insensitive HCC cells with the pan-ErbB family inhibitor Afatinib rather than with Erlotinib blocking ErbB1 reduced cell viability and clonogenicity. Genetic intervention with ErbB2-4 but not ErbB1 confirmed their crucial involvement in refractoriness to Rego. Furthermore, Rego-resistant HCC cells secreted basic fibroblast growth factor (bFGF) depending on Axl expression. HCC patients treated with Sora in first-line and with Rego in second-line displayed elevated serum levels of bFGF, emphasizing bFGF as a predictive biomarker of TKI treatment.

**Discussion:**

Together, these data suggest that the inhibition of ErbBs is synthetic lethal with Rego in Axl-expressing HCC cells, showing a novel vulnerability of HCC.

## 1 Introduction

Hepatocellular carcinoma (HCC) is the major subtype of liver cancer accounting for around 90% of cases. The incidence of HCC is expected to rise annually to over 1 million patients by 2025 (1). For HCC diagnosed at early stages, curative treatment options including surgical resection or liver transplantation are available but around 50% of HCC patients are diagnosed at advanced stages of the disease and receive systemic therapy (2). The most recently approved first-line treatment options are Durvalumab targeting programmed death ligand-1 (PD-L1) plus Tremelimumab targeting CTLA-4 or Atezolizumab (anti-PD-L1) in combination with Bevacizumab directed against vascular endothelial growth factor (VEGF) (3). HCC patients with autoimmune disease and transplant recipients are excluded from this treatment as they are at risk for severe immunological adverse events (1, 4, 5). Hence, these patients are subjected to first-line therapy with the tyrosine kinase inhibitors (TKIs) Sorafenib (Sora) or Lenvatinib (1). Additionally, the anti-VEGF treatment with Ramucirumab is approved as second-line treatment for HCC patients who progressed on first-line Sora, if serum α-fetoprotein (AFP) levels exceed 400 ng/mL (6). Together with Regorafenib (Rego) – which was the first approved second-line therapy of HCC – these drugs complement the spectrum of available treatment options for advanced stage HCC (7, 8). The acquisition of resistance against TKI treatment is a major challenge in HCC, which strongly limits the success of respective therapies. The overall survival (OS) of patients treated with Sora is roughly 3 months longer compared to placebo which emphasizes this limitation (9). Treatment with Rego in second-line increases the OS of patients progressing on Sora from 7.8 months to 10.6 months compared to placebo, highlighting the importance of investigating mechanisms of drug resistance and finding novel targets for treating advanced stage HCC patients (7). Several resistance mechanisms have been identified in different malignant settings including HCC, amongst them changes in epithelial plasticity by epithelial to mesenchymal transition (EMT). Cellular plasticity frequently confers insensitivity towards a panel of chemotherapeutic drugs in cancer models (10). Moreover, EMT is capable of conferring resistance towards epidermal growth factor receptor (EGFR) blockade in lung cancer and is linked to the induction of the receptor tyrosine kinase (RTK) Axl in HCC (11, 12). Axl is a driver of EMT and mediates resistance to cytotoxic drugs and TKIs in multiple cancer entities (13). Various mechanisms are involved including hetero-dimerization with other RTKs or decreased expression of miRNAs targeting Axl (14). In melanoma and triple-negative breast cancer, MEK inhibition led to increased activation of Axl and therefore increased tumor burden, while hetero-dimerization of Axl and Her2 reduced the response to Trastuzumab in Her2-positive breast cancer, highlighting the role of Axl in mediating resistance against targeted therapy (15, 16). In HCC, about 40% of patients show high levels of Axl, correlating with vascular invasion and poor survival of patients (17). Additionally, Axl has been identified as a driver of EMT and resistance to TKIs in HCC (18), underlining the potential of Axl as a mediator of resistance (11). In HCC cell lines, augmented expression of Axl has been reported upon acquiring resistance against Sora, which could be attenuated by targeting Axl (19, 20). Although multiple mechanisms of acquired Sora resistance in HCC have been investigated, studies focusing on Rego resistance are limited. The ErbB receptor family shows a wide range of functions including acquired drug resistance in a large number of cancer types (21). In HCC, activation of ErbB signaling allows liver cancer cells to escape from treatment with Lenvatinib (22). In non-small cell lung cancer resistant to Osimertinib, Axl associates with EGFR and ErbB3, suggesting a prominent role of Axl and ErbB coexpression in the refractoriness to targeted therapy resistance (23). In this study, we assessed the impact of Gas6/Axl on the response to TKI treatment in cellular HCC models. We observed that Gas6/Axl expression increases in TKI-resistant HCC cells exhibiting an EMT-transformed phenotype. Notably, Axl was associated with the upregulation and activation of ErbB receptors in Rego-resistant cells and with an elevated release of bFGF in HCC patients treated with Sora and Rego. These data suggest that co-activation of Axl and ErbB2-4 is crucially involved in acquiring resistance to Rego and that targeting ErbB receptors is a potential “Achilles heel” for intervention.

## 2 Material and methods

### 2.1 Cell culture

The human liver cancer cell lines SNU449 (RRID: CVCL_0454), SNU475 (RRID: CVCL_0497) and Huh7 (RRID: CVCL_0336) were propagated in RPMI-1640 supplemented with 10% fetal calf serum (FCS) and antibiotics. HLF (RRID: CVCL_2947) and HepG2 (RRID: CVCL_0027) cells were cultured in DMEM plus 10% FCS and 100 U/mL antibiotics. Hep3B cells (CVCL_0326) were cultured in EMEM supplemented with 10% FCS and antibiotics. SNU449-Axl^-^ cells were generated as previously described (24). SNU449, SNU475, Hep3B, HepG2 and Huh7 were purchased from ATCC (Manassas, VA, USA) and HLF and HLE cells were provided by Dr. Steven Dooley (University of Heidelberg, Germany). Cells were cultured at 37°C and 5% CO_2_. The identity of cell lines was confirmed by short tandem repeat analysis. All cell lines were routinely screened for the absence of mycoplasma.

### 2.2 Generation of TKI-resistant HCC cell lines

To generate resistant cell lines, SNU449, SNU449-Axl^-^, HLF and Huh7 were initially treated with 50% of the IC_50_ concentration of Sora (TargetMol Chemicals, Boston, USA), Rego (TargetMol Chemicals, Boston, USA), and Cabozantinib (Cabo; TargetMol Chemicals, Boston, USA). The concentrations of TKIs were increased by 50% of the IC_50_ every two weeks until 2 x IC_50_ was reached and cells proliferated under high concentrations.

### 2.3 Patient recruitment and sample collection

Patients were consecutively enrolled when treated with TKIs. Blood samples were obtained from patients with HCC who received Sora in first-line and Rego in second-line and sera were stored at -80°C. HCC was diagnosed according to the guidelines of the European Association for the Study of the Liver by histology or dynamic imaging such as computed tomography or magnetic resonance imaging scans (25). The study was conducted in accordance with the guidelines of the Declaration of Helsinki (1964, including current revisions) and after approval by the ethics committees of the Medical University of Vienna (EK2033/2017). All patients signed a written informed consent prior to study inclusion.

### 2.4 Enzyme-linked immunosorbent assay

In order to detect sAxl levels in cell culture supernatants, the human Axl ELISA (R&D Systems, Minneapolis, USA) was employed under optimized assay conditions as described (26). In order to analyze Gas6, the human Gas6 ELISA (R&D Systems, Minneapolis, USA) was used and included the replacement of Gas6 capture antibody with the engineered Axl decoy receptor AVB-S6-80 (Aravive Biologics, Houston, USA) (27). The ELISA for bFGF (R&D Systems, Minneapolis, USA) was carried out according to the manufacturer’s protocols. bFGF levels were determined in sera of treatment-naïve (n=24), first-line Sora treated (n=11) and first-line Sora/second-line Rego treated (n=9) HCC patients. In order to determine levels of Gas6 and sAxl, supernatants of respective cell lines were collected after cultivation in serum-free medium for 24 hrs. The supernatants were centrifuged to pellet remaining cells and stored at -80°C after snap-freezing until further use.

### 2.5 Proliferation kinetics

2 x 10^4^ cells were seeded into 12-well plates and counted on day 3, 5 and 7 each in triplicates. Before seeding, cells were pre-treated with the IC_50_ concentrations for 72 hrs of the respective TKI. Cell count was determined with the Casy Counter (Schärfe Systems, Reutlingen, Germany).

### 2.6 Cell viability assay

7.5 x 10^3^ cells per well were seeded into 96-well plates. After 24 hrs, cells were treated with increasing concentrations of Sora (TargetMol Chemicals), Rego (Cayman Chemical) and Cabo (TargetMol Chemicals), Afatinib (LC Laboratories) or Erlotinib (TargetMol Chemicals) and incubated for 72 hrs at 37°C and 5% CO2. After 72 hrs, 3-(4,5-Dimethylthiazol-2-yl)-2,5-diphenyltetrazoliumbromid (MTT) was added and incubated for 4 hrs at 37°C and 5% CO_2_. Next, the supernatant was aspirated and cells were lysed with DMSO for 1 hr at room temperature. The absorbance of the formazan was photometrically analyzed at 620 nm. The viability was calculated after subtraction of the blank values and normalized to untreated wells.

### 2.7 Clonogenicity

For the assessment of clonogenic growth behavior, 500 cells were seeded into 6-well plates in triplicates and either treated with increasing concentrations of Sora, Rego, Cabo, Afatinib or DMSO as a control. After colony formation, cells were washed, fixed with acetic acid/methanol solution (3:1), stained with 0.025% crystal violet and colonies counted.

### 2.8 Quantitative PCR

RNA from cells was isolated using the Monarch™ Total RNA Miniprep Kit (New England Biolabs, Ipswich, USA). RNA concentration was determined using Nanodrop (Thermofisher, Waltham, USA). 500 ng of RNA was reverse transcribed using the iScript™ cDNA synthesis kit (BioRad, Hercules, USA). For quantitative PCR (qPCR), the LUNA Universal qPCR Master Mix (New England Biolabs, Ipswich, USA) and the CFX Connect Real Time PCR system (BioRad, Hercules, USA) was employed. Following primers were used: *RPL41*: forward 5’-CAAGTGGAGGAAGAAGCGA-3’, reverse 5’-TTACTTGGACCTCTGCCTC-3’; *AXL*: forward 5’-CCGGCTGGCGTATCAAGGCC-3’, reverse, 5’-TGGCTGTGCTTGCCCTGGG-3’; *EGFR*: forward 5’-GTGAACCCCGAGGGCAAATA-3’, reverse 5’-ATTCCGTTACACACTTTGCGG-3’; *ErbB2*: forward 5’-TCCTCCTCGCCCTCTTGC-3’, reverse 5’-AGTTCCAGGTTTCCCTGCAC-3’; *ErbB3*: forward 5’-GGGACCGAGATGCTGAGATA-3’, reverse 5’-GCCCAAAGCAGTGACCATTA-3’; *ErbB4*: forward 5’-CGGGCCATTCCACTTTACCA-3’, reverse 5’-GAGCTTGATTGGGTGCTGTG-3’.

### 2.9 RNA-Seq

3’-end mRNA libraries were generated with QuantSeq and subjected to Illumina sequencing with the HiSeq 2500 system by Lexogen (12 cycles of library amplification, paired-end 100 nt sequencing). Computational trimming of poly-As from the 3’-ends of the 100 bp long reads was performed with cutadapt (v. 4.0; with options -O 3 -e 0.05 -m 20 –max-n 2), retaining reads with a trimmed length of at least 20 nt. Reads were aligned to the human genome GRCh38.v40 and the spike-in sequences (Lexogen “SIRV-Set3”) by STAR 2.5.3a (28), with up to 1000 hits, a maximum error rate of 5%, and counts per gene as output. These count tables were generated on the basis of the GENCODE annotation v40 concatenated with the SIRV and ERCC annotation. For visualizing potential batch effects, normalized samples were clustered hierarchically (R hclust, stats package), or analyzed by principal component analysis and pair-wise plotting of the first five principal components (PCA, package FactoMineR). Pair-wise differential expression analyses were performed with DESeq2 (latest version downloaded from Bioconductor v. 3.15) (29). Low-expressing genes with less than 3 counts in at most 3 samples were filtered out. Moreover, duplicate gene ids were removed by retaining the highest expression levels, respectively, resulting in a final set of 15,633 genes. DESeq2 default parameters were used except for the dispersion model fitting. Moreover, the preparation dates were included in the design formula in order to correct for this technical bias. In order to obtain batch-corrected values for heat map visualisation and gene set enrichment analysis (GSEA), log-vst transformation (DESeq2) and limma *removeBatchEffect* correction were performed. The heat map was created with a customized R script based on heatmap.2.R, with row-wise z-score scaling. All reported p-values (including cut-offs) for differential expression analyses refer to Benjamini-Hochberg multiple-test corrected q-values, as implemented by DESeq2. For GSEA, the command-line version 4.3.1. of the UCSD/BROAD GSEA tool (30) was used, with Diff_of_Classes ranking (to account for the log-transformed count values) and gene-set permutation. Duplicate gene symbols were removed based on their rank. Gene sets of Biocarta, CGP, GO, Hallmark, Kegg, ONCO, PID, and Reactome v2022 were downloaded from the Molecular Signatures Database (MSigDB) and filtered for a set size of 30 – 500 genes. If not stated otherwise, analyses were performed using custom R scripts and RStudio.

### 2.10 RTK array

Parental (control), TKI-resistant HCC cells and TKI-resistant HCC cells lacking Axl were analyzed for the activation of RTKs by using the Proteome Profiler Human Phospho-RTK Array (R&D Systems, Minneapolis, USA) as published. The phospho-RTK array contains 49 different RTK antibodies spotted in duplicates. In brief, antibody arrays were blocked and incubated with 300 µg of whole cell lysates overnight at 4°C. After washing, membranes were supplemented with an anti-phospho-tyrosine-HRP antibody and incubated for 2 hrs at room temperature. After washing, arrays were incubated with a chemiluminescent substrate and exposed to x-ray films for different time points. Spots were analyzed using Image Studio Lite™ (LI-COR Biosciences, Lincoln, USA) and normalized to their respective control spots.

### 2.11 Growth factor array

Supernatants of parental (control), TKI-resistant HCC cells and TKI-resistant HCC cells lacking Axl were analyzed for secretion of growth factors by using the Human Growth Factor Array (Abcam, Cambridge, UK) which allows for the identification of 41 growth factors. After blocking of the antibody arrays, supernatants of naïve and Rego-resistant SNU449 and SNU449-Axl^-^ cells were incubated with the arrays at 4°C overnight with slight agitation. After incubation, arrays were washed and incubated with anti-phospho-tyrosine-HRP for 2 hrs at room temperature. Arrays were further washed and incubated with a chemiluminescent substrate and developed on x-ray films using different time points. Spot intensities were analyzed using Image Studio Lite™ (LI-COR Biosciences, Lincoln, USA) and normalized to control spots.

### 2.12 siRNA-mediated knockdown

Cells were transfected with small interfering RNAs (siRNA; Dharmacon, Lafayette, USA) against Axl, EGFR, ErbB2, ErbB3 and ErbB4 as described in (17). For the MTT assay, 7.5 x 10^3^ cells were each seeded into 96-well plates and transfected with 50 nM of respective siRNA using Lipofectamine (Invitrogen, Waltham, USA) according to the manufacturer’s protocol. After adherence of cells, increasing concentrations of Rego were added to the wells and incubated for 72 hours at 37°C and 5% CO2. For the detection of knockdown efficiency, 1 x 10^5^ cells were seeded into 12-well plates and transfected with respective siRNAs. Cells were harvested after 72 hrs for RNA extraction and qPCR.

### 2.13 Western blot

Immunoblotting was done as described (31). In brief, blotted membranes were blocked in 4% bovine serum albumin (BSA) in TBS-T (0.1%). The primary anti-Axl (R&D Systems; #AF154) and anti-actin (Sigma; #A2066) were diluted 1:1000 in blocking solution and incubated overnight at 4°C.

### 2.14 Immunofluorescence

SNU449 and SNU449-Axl^-^ cells were stained for Axl and analyzed as previously described (17). In brief, cells were seeded onto collagen coated coverslips for 24 hrs. After the cells have adhered, they were fixed in 4% phosphate-buffered formaldehyde at 37°C. After washing cells with PBS, cells were blocked with 5% horse serum in 1% BSA/PBS for 1hr at room temperature. Axl antibody (R&D Systems; #AF154) was diluted 1:1000 in blocking solution and incubated for 1 hr at room temperature. Subsequently, the cells were stained with an anti-goat Alexa Fluor 488 tagged antibody (Biotrend; #20225-1) at 1:200 in blocking solution. Finally, nuclei were stained with DAPI at a dilution of 1:1000 for 10 min. Cells were mounted with mowiol and imaged with the Zeiss Confocal LSM 700 (Carl Zeiss Microscopy GmbH; Jena, Germany).

### 2.15 Statistical analysis

Data are expressed as mean +/- standard deviation (SD). Normally distributed data between two groups were analyzed via Student’s t-test. For statistical analysis, we used GraphPad Prism 5.02 (GraphPad Software Inc, San Diego, USA). P values were considered as statistically significant as follows: ns p>0.05 *p<0.05; **p<0.01; ***p<0.001.

## 3 Results

### 3.1 TKI resistance correlates with EMT and Axl expression

**Figure 1:**
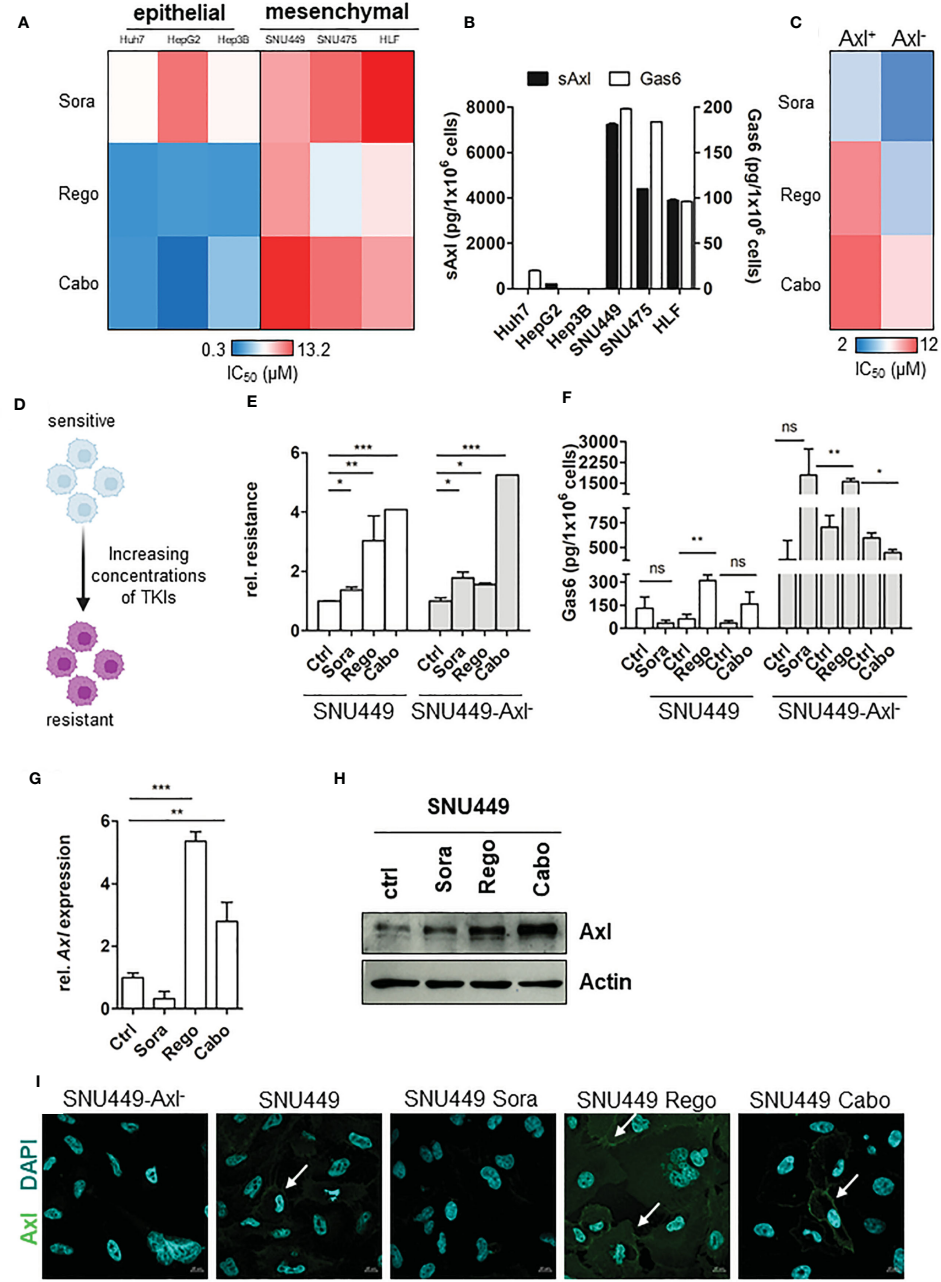
Axl modulates cellular response to TKIs. **(A)** Heat map of IC_50_ values showing sensitivities of HCC cells against TKIs. **(B)** Release of sAxl and Gas6 into supernatants of HCC cells as determined by ELISA. **(C)** Heat map of IC_50_ values indicating sensitivities of parental and Axl-deficient HCC cells against TKIs. **(D)** Model depicting the generation of TKI-resistant cells. **(E)** Relative response of parental (ctrl, control, naïve) and resistant HCC cells against TKIs in Axl-proficient and Axl-deficient background. IC_50_ values were calculated and normalized to sensitive control. Sensitivity of the control was set to the value of 1. **(F)** Release of Gas6 into supernatants of control (naïve) and TKI-resistant cells as determined by ELISA. **(G, H)** Transcript and protein levels of Axl in control (naïve) and TKI-resistant HCC cells as analyzed by qPCR **(G)** and Western Blotting **(H)**, respectively. **(I)** Expression of Axl (green) in control (naïve) and Sora-, Rego- and Cabo-resistant HCC cells as examined by immunofluorescence microscopy. Cell nuclei were stained with DAPI (blue). White arrows show Axl expression on cell membranes. Data are expressed as mean +/- SD. ns: p > 0.05; *p ≤ 0.05; **p ≤ 0.01; ***p ≤ 0.001. TKI, tyrosine kinase inhibitor; Sora, Sorafenib; Rego, Regorafenib; Cabo, Cabozantinib; sAxl, soluble Axl.

To investigate mechanisms of resistance to therapy in HCC, we analyzed the sensitivity of epithelial and mesenchymal-like liver cancer cells against the TKIs Sorafenib (Sora), Regorafenib (Rego) and Cabozantinib (Cabo). Importantly, all selected cell lines are not classified as intrinsically resistant based on sensitivity screens towards a large spectrum of therapeutic agents as described by Qiu and colleagues (32). While all cell lines responded to treatment, we observed that the mesenchymal-like HCC cell lines SNU449, SNU475 and HLF were more tolerant in comparison to epithelial Huh7, HepG2 and Hep3B cells, as indicated by their respective half maximal inhibitory concentration (IC_50_; **Figure 1A**). As Axl and its cleavage product soluble Axl (sAxl) have been described to be elevated in EMT-transformed hepatoma cells (17), we examined sAxl and Gas6 levels in corresponding supernatants and found a highly elevated release of both in mesenchymal-like HCC cells (**Figure 1B**). Hence, we investigated whether decreased sensitivities of EMT-transformed HCC cells against TKIs depend on Axl expression. Interestingly, cells lacking Axl after CRISPR/Cas9-mediated genomic editing were more sensitive to TKIs supporting the hypothesis that Axl favors tolerance against targeted therapies in HCC (**Figure 1C**). To study the role of Axl in losing sensitivity to TKIs, we generated a model of stable resistance by treating SNU449 and SNU449-Axl^-^ cells each with increasing concentrations of either Sora, Rego or Cabo (**Figure 1D**). Determination of IC_50_ values of SNU449 and SNU449-Axl^-^ cells upon respective TKI treatment revealed that these cells were indeed less sensitive compared to untreated control, suggesting them as valuable tools to study Axl-dependent mechanisms of resistance in HCC (**Figure 1E**). SNU449 and SNU449-Axl^-^ cells - both resistant to Sora, Rego or Cabo - showed increased clonogenic growth behavior in the presence of the respective TKI, confirming that these cells indeed resist higher concentrations of the TKIs compared to their naïve controls (**Figures S1A, B**). However, proliferation was attenuated in the absence of drugs except for Cabo-resistant SNU449 cells (**Figure S1C**). As we observed an increased number of colonies at lower proliferation rates, the TKI-resistant cell lines show phenotypic changes resembling cancer stem cells or drug persister cells which are characterized by self-renewal at a slow-cycling states (33). Interestingly, Gas6 and Axl levels were increased in Rego- and Cabo-resistant HCC cells (**Figures 1F–I**). In particular, Axl expression was elevated upon acquiring resistance to Rego, both on transcript and protein levels (**Figures 1G, H**). In addition, high Axl expression was strongly displayed at cell boundaries in Rego- rather than in Sora and Cabo-resistant cells (**Figure 1I**). Together, these data suggest that Axl has a key role in acquiring resistance against the clinically used TKIs in HCC.

### 3.2 ErbB2-4 expression depends on Axl in resistance to Rego

**Figure 2:**
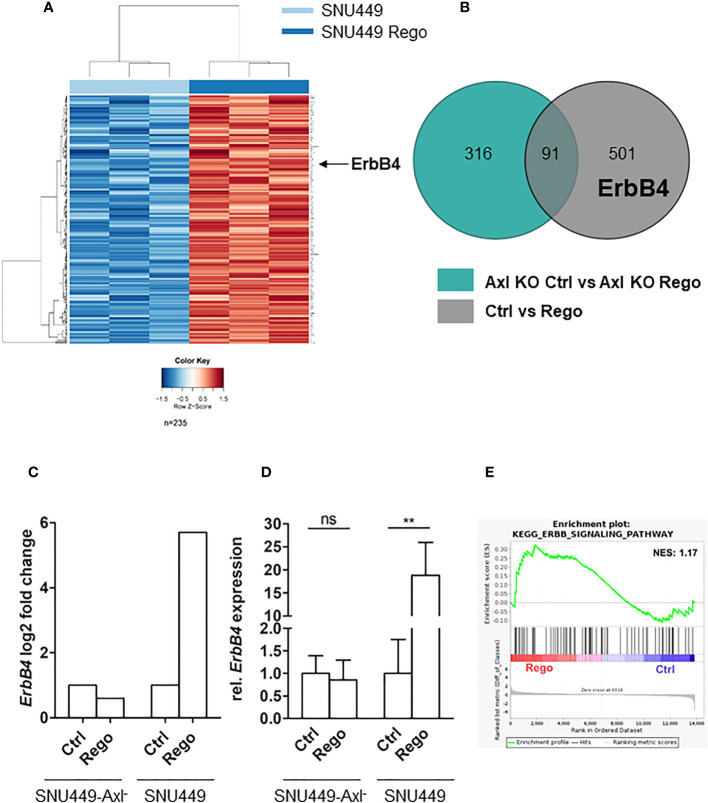
Axl expression associates with induction of ErbB4 levels in Rego-resistant HCC cells. **(A)** Heat map of differentially expressed transcripts in control (naïve; light blue bar) vs. Rego-resistant SNU449 cells (dark blue bar). Heat map colors range from blue to red for low to high relative expression (see color gradient at bottom). Arrow indicates the row showing ErbB4 expression in Rego-resistant HCC cells. **(B)** Axl-dependent genes associating with Rego resistance were identified by overlapping differentially expressed genes of control vs Rego-resistant cell comparisons in an Axl-deficient (green) or Axl-proficient (grey) background. **(C)** Log_2_FC of ErbB4 expression in Rego-resistant versus control cells based on RNA-Seq data. **(D)** Relative mRNA expression of *ErbB4* in control and Rego-resistant SNU449 and SNU449-Axl^-^ as determined by qPCR. Data are expressed as mean +/- SD. **(E)** GSEA shows enrichment of ErbB signaling in Rego-resistant SNU449 cells. ns: p > 0.05; **p ≤ 0.01. GSEA, Geneset enrichment analysis; Rego, Regorafenib; FC, fold change.

**Figure 3:**
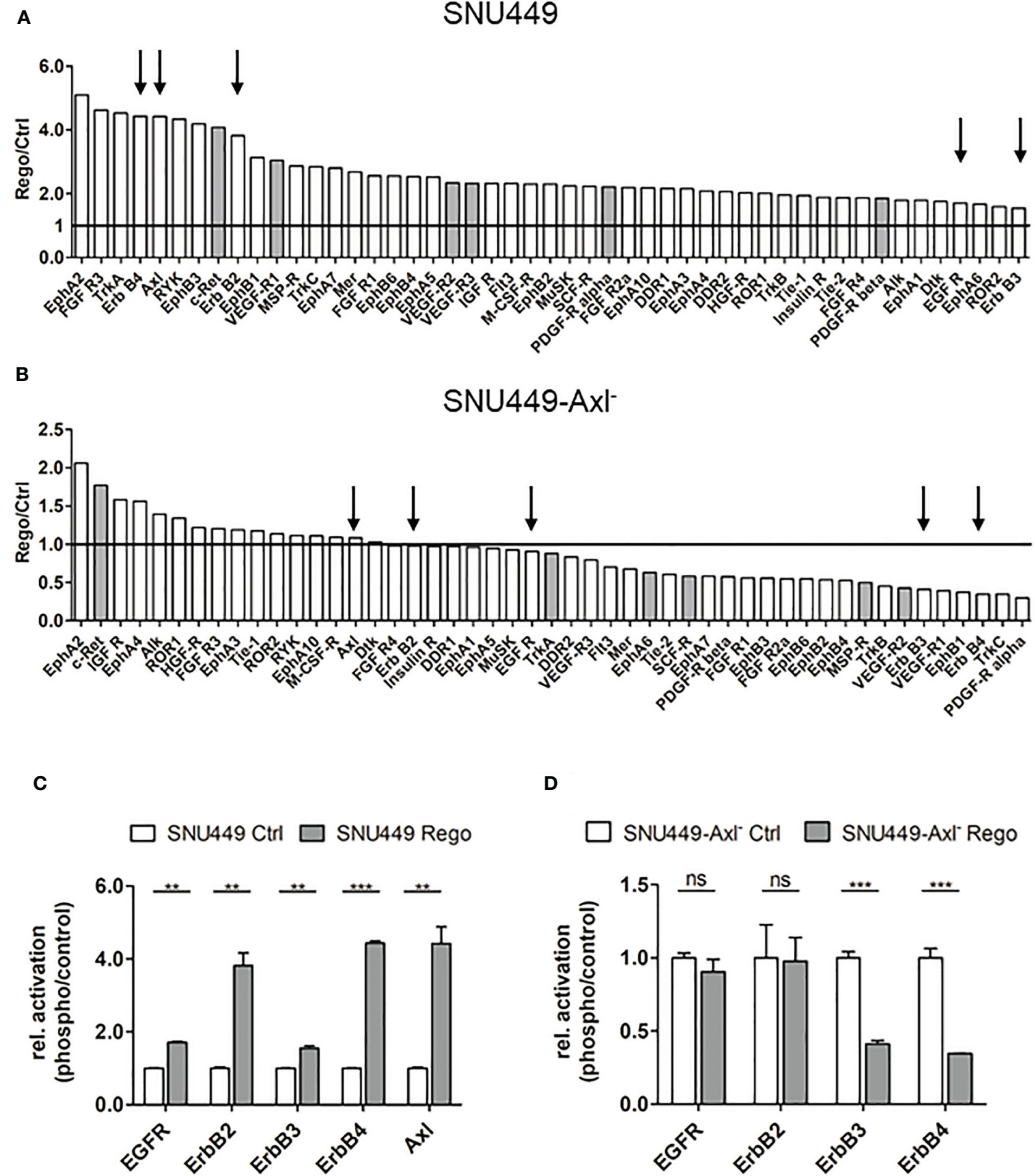
Axl-dependent activation of ErbB receptors in Rego-resistant HCC cells. **(A, B)** Quantification of phospho-RTK signals comparing control (ctrl, naïve) and Rego-resistant SNU449 cells in the Axl-proficient **(A)** and Axl-deficient background **(B)**. Signal intensities of phospho-RTKs were normalized to intensities of controls. Grey bars depict targets of Regorafenib. Arrows indicate Axl and members of the ErbB receptor family and black lines indicate unchanged phosphorylation levels **(A, B)**. **(C, D)** Relative abundance of selected phospho-RTKs in control (naïve) vs. Rego-resistant SNU449 cells in the Axl-positive **(C)** and Axl-negative background **(D)**. Data are expressed as mean +/- SD. ns: p > 0.05; **p ≤ 0.01; ***p ≤ 0.001. RTK, receptor tyrosine kinase; Rego, Regorafenib.

Since Axl expression was strongly elevated both on transcript and protein level and correlated with increased Gas6 levels in Rego-resistant SNU449 (**Figures 1F, G–I**), we were interested in examining the involvement of Axl in acquired Rego-resistance of HCC cells. In a first approach, we performed RNA-seq analysis of parental versus Rego-resistant cells in an Axl-proficient and Axl-deficient background. Differential gene expression analysis revealed 235 upregulated genes in Rego-resistant SNU449 cells compared to control (**Figure 2A**). Next, we filtered the significant differentially expressed genes to get a specific Axl-dependent and Rego-resistance-associated set of genes. Interestingly, we observed that the upregulation of ErbB4 upon acquiring resistance to Rego depended on Axl expression (**Figures 2B, C**). In independent experiments, we verified these results and observed an 18.8-fold increase of ErbB4 transcript levels of SNU449-Rego cells compared to control (**Figure 2D**), while SNU449-Axl–Rego cells showed no differential expression. In this line, genes correlating with ErbB signaling were enriched in Rego-resistant SNU449 (**Figure 2E**). To further investigate whether elevated ErbB4 expression correlates with ErbB4 activation, we analyzed the phosphorylation of ErbB receptors and Axl by phospho-RTK analysis. Accordingly, we observed increased activation of all ErbB receptors in Rego-resistant, Axl-proficient SNU449 cells compared to control (**Figures 3A, C**, **S2**). Both ErbB4 and Axl displayed a more than 4-fold elevated phosphorylation in Rego-resistant cells. Interestingly, no changes in activation (EGFR, ErbB2) or even a reduced phosphorylation of ErbBs (ErbB3, 4) were observed in the Axl-deficient background (**Figures 3B, D**, **S2**). These results show that the upregulation of ErbB4 mRNA and the augmented activation of ErbBs depend on Axl expression in Rego-resistant HCC cells.

### 3.3 Pharmacological intervention with Afatinib re-sensitizes Rego-resistant HCC cells

**Figure 4:**
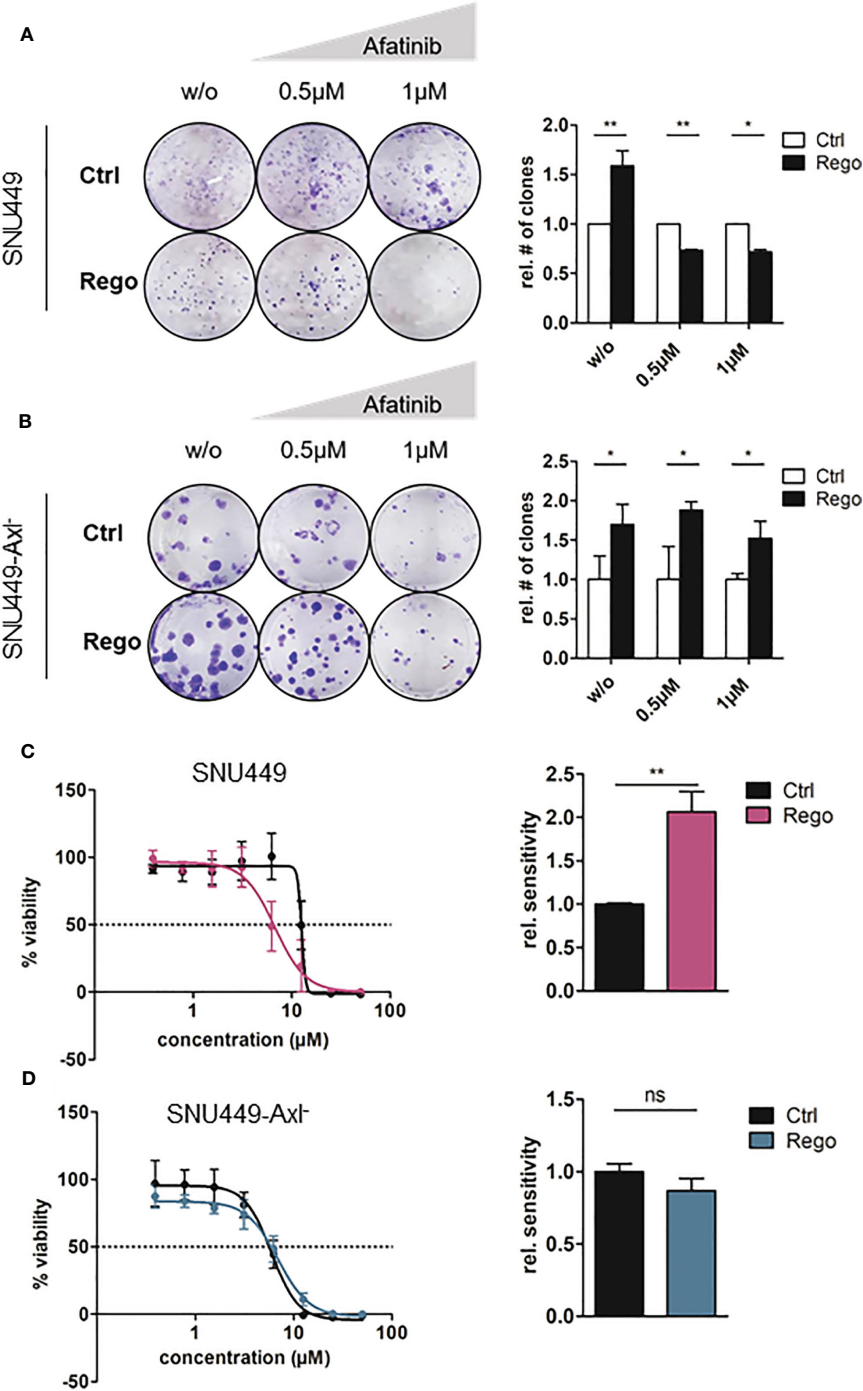
Resistance to Rego sensitizes Axl-expressing cells towards Afatinib. **(A, B)** Clonogenic growth behavior of control (naïve) and Rego-resistant SNU449 cells untreated (w/o) or treated with increasing concentrations of Afatinib in the Axl-proficient **(A)** and Axl-deficient background **(B)**. Bar charts depict the quantification of colonies relative to control (right panels). **(C, D)** Dose-response curves of control and Rego-resistant SNU449 cells against Afatinib in the Axl-positive **(C)** and Axl-negative background **(D)**. Bar charts show sensitivity (IC_50_) against Afatinib relative to controls **(C, D)**. **(A–D)** Sensitivities of controls were set to the value of 1. Data are expressed as mean +/- SD. ns: p > 0.05; *p ≤ 0.05; **p ≤ 0.01; Rego, Regorafenib.

Given the high activation levels of ErbB1-4 in Rego-resistant HCC cells, we wanted to address whether these cells rely on ErbB signaling. Afatinib is an irreversible, second-generation pan-ErbB inhibitor which is currently used in patients with local and metastatic non-small cell lung cancer (34). Thus, we tested whether the treatment with Afatinib or Erlotinib, a first-generation inhibitor of EGFR, could re-sensitize them to treatment with Rego. Resistant and naïve SNU449 or SNU449-Axl^-^ cells were treated with increasing doses of Afatinib or Erlotinib and clonogenic growth behavior as well as IC_50_ values were determined. Afatinib abrogated clonogenic growth behavior of Rego-resistant SNU449 cells in a dose-dependent manner but did not affect naïve SNU449 cells (**Figure 4A**). Notably, the reduced clonogenicity upon Afatinib was dependent on Axl as these differences were not observed in SNU449-Axl^-^ cells irrespectively of the resistance against Rego (**Figure 4B**). Importantly, we observed that Rego-resistant cells were significantly more sensitive to Afatinib than the naïve control indicating a dependency on ErbB receptors (**Figure 4C**). In contrast, Afatinib did not re-sensitize Rego-resistant SNU449-Axl^-^ cells (**Figure 4D**) confirming that elevated ErbB activation is associated with Axl expression in Rego-resistant HCC cells. Comparable results were obtained by analyzing Rego-resistant, Axl-proficient HLF and epithelial Huh7 cells, which express low levels of Axl (**Figures S3A–D**). Notably, inhibition of EGFR on its own by treatment of cells with Erlotinib did not increase the sensitivity of SNU449 or SNU449-Axl^-^ against Rego (**Figures S3E–H**). From these data we concluded that inhibition of ErbB2-4, but not EGFR, confers sensitivity to Rego in Axl-proficient HCC cells.

### 3.4 Genetic intervention with ErbB2-4 re-sensitizes HCC cells to Rego

**Figure 5:**
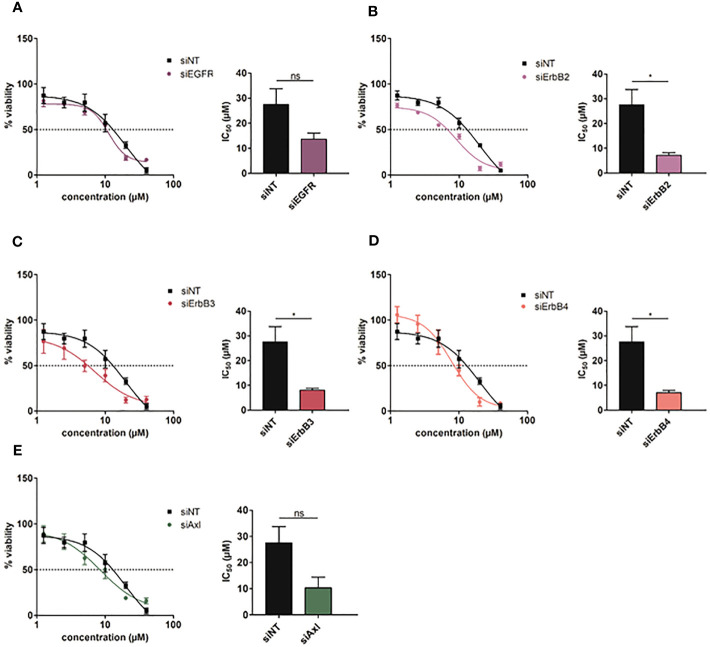
Intervention with ErbB2-4 expression re-sensitizes Rego-resistant HCC cells. **(A-E)** Dose-response curves of Rego-resistant SNU449 cells against Regorafenib upon siRNA-mediated knockdown of *EGFR* **(A)**, *ErbB2* **(B)**, *ErbB3* **(C)**, *ErbB4* **(D)** and *Axl* **(E)**. Bar charts show the respective IC_50_ values (µM). Data are expressed as mean +/- SD. ns: p > 0.05; *p ≤ 0.05. NT, Non-Target.

To investigate which ErbB receptor is crucially involved in conferring resistance to Rego, we performed knockdowns of each ErbB and treated refractory cells with increasing doses of the TKI. We assumed that abrogating the expression of ErbBs is essentially involved in mediating resistance and should cause re-sensitization to Rego. Downregulations of EGFR, ErbB2, ErbB3 and ErbB4 after siRNA applications were verified by qPCR (**Figure S4A**). Interestingly, we observed that knockdown of ErbB2, ErbB3 and ErbB4 rather than EGFR significantly restored the sensitivity to Rego compared to the non-target control in Rego-resistant SNU449 cells (**Figures 5A–D**). Yet, knockdowns of ErbBs in naïve SNU449 cells did not alter the response to Rego (**Figures S4B–F**). Notably, knockdown of Axl in Rego-resistant SNU449 cells induced a trend towards augmented sensitivity to Rego, yet to a weaker extent than the knockdown of ErbB2-4 (**Figure 5E**). Together, these data confirm that ErbB2-4 are involved in the resistance to Rego in HCC cells.

### 3.5 Resistance to Rego induces bFGF secretion

**Figure 6:**
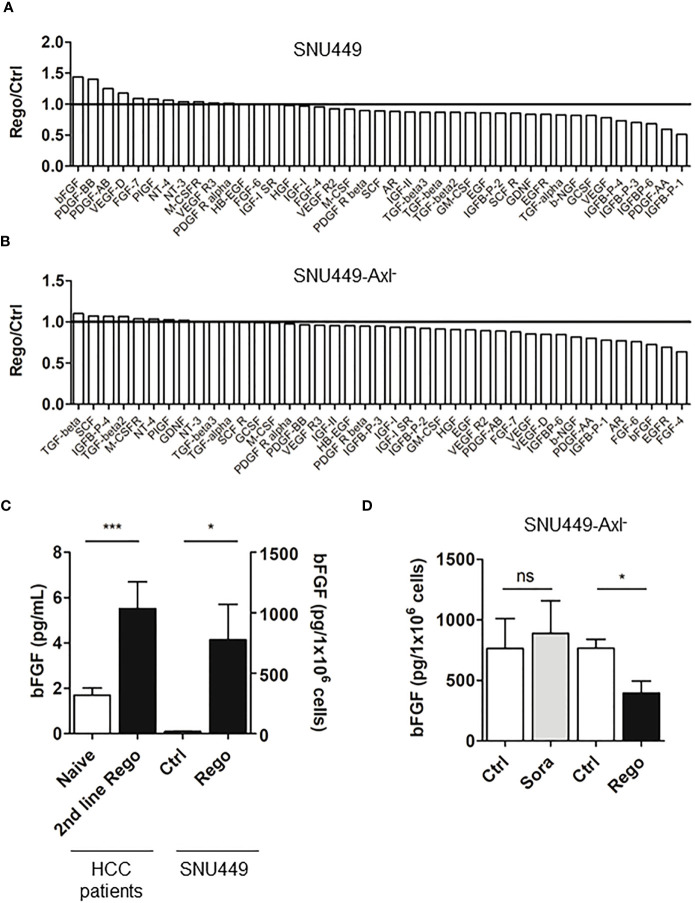
Resistance to Rego induces bFGF secretion. **(A, B)** Quantification of growth factor signals comparing control (ctrl, naïve) and Rego-resistant SNU449 cells in the Axl-proficient **(A)** and Axl-deficient background **(B)**. Bar charts depicts relative abundance of growth factors to their respective naïve control. Black lines indicate unchanged growth factor levels **(A, B)**. **(C)** Levels of bFGF in sera of naïve and 2^nd^ line Rego-treated HCC patients and supernatants of control (white) and Rego-resistant SNU449 cells as determined by ELISA. **(D)** bFGF levels in supernatants of control (white) and Sora- and Rego-resistant SNU449-Axl^-^ cells (grey, black) as determined by ELISA. Data are expressed as mean +/- SD. ns: p > 0.05; *p < 0.05. ***p ≤ 0.001. Rego, Regorafenib; Sora, Sorafenib.

We further concentrated on the opportunity to identify biomarkers indicating responsiveness of HCC patients to treatment with Rego. By detecting growth factors using antibody arrays, we found that Rego-resistant SNU449 cells secreted 1.44-fold more bFGF compared to the naïve control (**Figures 6A**, **S5A**). Of note, resistance to Rego did not significantly induce the secretion of bFGF in Axl-deficient SNU449 cells (**Figure 6B**), suggesting that bFGF secretion is linked to Axl expression. By analyzing whether the secretion of bFGF was dependent on the resistance to Rego, we found a significant increase of bFGF in supernatants of Rego- rather than in Sora-resistant HCC cells by ELISA (**Figure 6C**, right panel; **Figure S5B**, right panel). Additionally, we confirmed that the secretion of bFGF is increased in Rego-resistant cells expressing Axl (**Figure 6C**, right panel) but fails to be upregulated in Axl-deficient cells (**Figure 6D**). To translate data from cellular models to HCC patients, we determined serum levels of bFGF in treatment naïve patients, in patients exclusively treated first-line with Sora and in patients treated first-line with Sora and second-line with Rego (**Table S1**). Remarkably, we observed increased levels of bFGF in sera of patients treated first-line with Sorafenib (**Figure S5B**, left panel) and in sera of patients treated second-line with Rego compared to treatment-naïve patients (**Figure 6C**, left panel). Together, these data suggest that bFGF levels are augmented in a Rego-resistant cell model correlating with Axl and in HCC patients treated with Rego in second-line.

## 4 Discussion

Refractoriness to TKIs is the major cause of systemic treatment failure in HCC patients. In the current study we show that changes in epithelial plasticity impact TKI response as EMT of HCC cells is associated with reduced drug sensitivity when compared to epithelial cells. Yet, all determined IC_50_ values were within the lower µM range, indicating response to the administered inhibitors and in line with previously published data (32). Abrogation of the response to TKIs was further accompanied by the upregulation of Gas6 and Axl levels, most prominently in Rego-resistant HCC cells. Elevated Axl abundance was closely linked to the augmented expression and activation of ErbB receptors. Addiction of Rego-resistant cells to ErbB receptors was validated by pharmacological intervention, revealing an increased susceptibility to the pan-ErbB inhibition by Afatinib. In accordance, genetic interference with ErbB2/ErbB4 re-sensitized resistant HCC cells to Rego. Moreover, HCC cells resistant to Rego secreted enhanced levels of bFGF, which were recapitulated in sera of first and second-line treated HCC patients.

EMT can be induced through multiple tumor cell-intrinsic and extrinsic factors and is crucially involved in various pathophysiological events including drug resistance (35). Human HCC cells have been reported to upregulate mesenchymal markers such as Snail1 and Vimentin upon the acquisition of resistance to Rego (36). Furthermore, EMT has been associated with reduced sensitivity of HCC cells to Sorafenib emphasizing the key role of cellular plasticity in TKI resistance (37–40). Disintegration of epithelial organization by the dissociation of E-cadherin/β-catenin complexes results in the nuclear translocation of β-catenin which promotes the resistance to Sora in HCC (41). In line, we found that HCC cells harboring an EMT phenotype tolerate higher concentrations of the TKIs Sora, Rego and Cabo as compared to epithelial HCC cells. Notably, Axl was recently described as one of the main regulators of EMT in HCC and other cancer entities modulating the response to kinase inhibitors (11, 17, 42). Accordingly, Gas6 secretion was strongly increased in Rego-resistant HCC cells together with elevated Axl transcript and protein expression. Unexpectedly, Gas6 levels were highly abundant in Rego-resistant cells lacking Axl. We hypothesize that these elevated levels in Axl-deficient cells stem from unbound Gas6 in cell supernatants. Interestingly, we observed that Axl and Gas6 are stronger expressed in Rego-resistant HCC cells as compared to Sora-resistant cells. While Axl levels did not alter in Sora-resistant cells, we observed increased phosphorylation of Axl (data not shown), suggesting that Axl signaling is involved in mediating resistance to Sorafenib as described previously (20).

Rego-resistant HCC cells exhibited upregulation of Axl together with augmented expression and activity of the ErbB receptor family, suggesting a molecular collaboration of these RTKs. Noteworthy, Tyro3 and MerTK were not upregulated in Axl-deficient HCC cells, suggesting that no compensatory mechanisms are provided by these members of the TAM receptor family. In breast cancer, the heterodimerization of ErbB2 and Axl induces EMT and therapy resistance by regulating Akt and Mek signaling (15). Another study has shown that ErbB2 and Axl heterodimerize and that downstream activation depends on trans-phosphorylation by ErbB2 but not Gas6 (43). Moreover, Axl confers resistance to EGFR-targeted therapy in lung cancer by interacting with ErbB3 and phosphorylation of Akt (23). In our study, the impact of Rego-resistant cells on ErbB receptors was validated by their increased sensitivity to Afatinib. Accordingly, genetic ablation of ErbB2-4 re-sensitized them to Regorafenib. In HCC, a CRISPR-Cas9-based screening revealed that EGFR is synthetic lethal with Lenvatinib, thus blocking of fibroblast growth factor receptor (FGFR), a target of Lenvatinib, leads to upregulation of EGFR (22). These findings have been supported by other groups showing increased EGFR expression activating the STAT3-ABCB1 pathway in Lenvatinib-resistant HCC cells (44). Noteworthy, ErbB2 and ErbB3 were activated in HCC cells upon acquiring resistance to pan-FGFR inhibition (45). These findings allow to speculate that the activation of ErbB2 and ErbB3 in Rego-resistant HCC cells is due to the inhibition of FGFR, which is a target of Rego. Yet, based on our RNA-Seq and qPCR data, we found that ErbB4 displayed the highest expression linked to Axl in Rego-resistant cells which is a novel finding in HCC. Unfortunately, we could not detect Axl/ErbB4 co-expression via immunofluorescence analysis as various antibodies did not allow to reliably determine ErbB4 expression. Further studies will focus on the synergy of Axl and ErbB receptors in HCC and will analyze potential physical interactions including downstream signaling events.

Another challenge in HCC therapy is the absence of predictive biomarkers which allow to select the best treatment at advanced stage of disease and to surveil the response to therapy (2). The modulation of AFP upon TKI therapy is the most frequently used predictive biomarker in HCC patients, together with PD-L1 expression, tumor lymphocyte infiltration and immune class signatures (46). We detected elevated bFGF levels in sera of patients treated with frontline Sora and second line Rego as well as in supernatants of Axl-proficient HCC cells resistant to Rego. Interestingly, the secretion of bFGF depends on Axl expression (**Figures 6A, B**) rather than on the synergism of Axl and ErbBs as treatment of Rego-resistant HCC cells with Afatinib did not affect the release of bFGF into supernatants (data not shown). FGFR1 transcript levels are elevated in HCC patients at advanced stages and bFGF is able to induce PD-1 expression on T cells, therefore limiting their functionality (47). Furthermore, FGFR1 signaling is elevated in Sora-resistant HCCs, highlighting our findings that increased bFGF levels are not restricted to HCC patients treated with Rego (48). bFGF is described to be upregulated upon anti-angiogenic treatment by VEGF inhibition, which could explain the increased levels upon treatment with Sora and Rego as these TKIs target VEGFR2-3 and VEGFR1-3, respectively (49). These findings implicate that patients could benefit from synergistic targeting of VEGFR and FGFR-signaling to overcome resistance. Together, these findings support our hypothesis that inhibition of FGFR and VEGFR by treatment with Rego induces bFGF secretion which represents a predictive biomarker in Rego-resistant HCC cells but not in Sora-resistant ones. Noteworthy, Axl is a downstream target of hypoxia-inducible transcription factor-1 (HIF-1) and HIF-2 in hypoxic cancer cells (50). Treatment with Bevacizumab causes bFGF and HIF-2 expression indicating a potential link between Axl and bFGF (51).

Combinatorial treatment of Atezolizumab and Bevacizumab is the novel first line therapy regimen for patients with advanced HCC (4). However, HCC patients with comorbidities that require treatment with immunosuppressants rely on treatment with TKIs. Therefore, it is still relevant to understand the resistance mechanisms underlying TKI treatment in order to ensure proper care of these patients. We found that the FDA-approved pan-ErbB inhibitor re-sensitized Rego-resistant HCC cells. Unfortunately, we cannot conclude whether Axl mediates the upregulation of ErbB receptors or whether Axl and ErbBs physically interact to induce resistance. Hence, further studies are required to investigate signaling pathways inducing resistance to Rego in HCC.

Due to the increased risk of severe complications and lack of therapeutic advantage, advanced HCC patients are not subjected to tissue biopsy. Thus, our study lacks the confirmation of ErbB expression in Rego-treated HCC patients. Moreover, treatment of Rego-resistant tumors with Afatinib after xenotransplantation would provide further evidence whether inhibition of ErbB receptors reduces tumor growth. However, the cell lines used in this study failed to engraft in immunocompromised mice. In addition, a larger number of HCC serum samples is required to strengthen our data and to investigate whether bFGF could serve as a predictive marker for Sora- and Rego-resistance. As an increasing number of patients receive immunotherapy rather than TKIs, the number of Sora-treated (n=11) and Rego-treated (n=9) HCC patients is limited.

**Figure 7:**
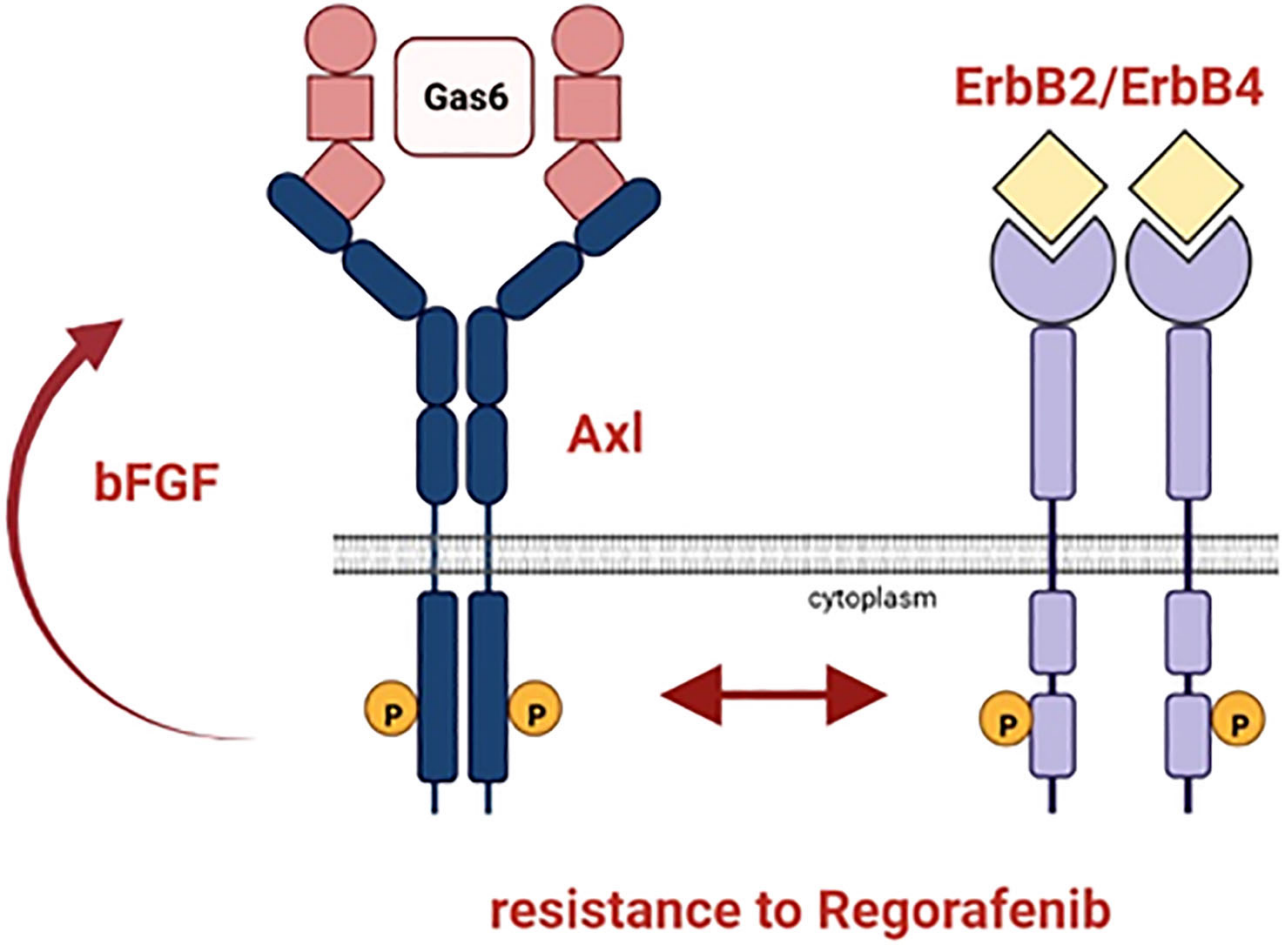
Schematic representation of the resistance mechanism of HCC cells to Rego. Axl and ErbB2-4 are co-upregulated in HCC cells resistant to Rego. bFGF levels increase upon acquiring resistance to Rego.

In summary, we observed that the ErbB receptor family, especially ErbB4, is co-upregulated with Gas6/Axl in Rego-resistant HCC cells indicating an Axl/ErbB receptor-mediated therapy escape (**Figure 7**). Accordingly, the HCC models are highly sensitive towards ErbB receptor blockade by Afatinib and genetic intervention with the ErbB2-4 re-sensitized cells to Rego. Additionally, we found that bFGF is elevated in sera of Sora- and Rego-treated patients. We conclude that the synergy between Gas6/Axl and the ErbBs, especially ErbB4, could serve as a potential target for patients who progressed on targeted therapy with Rego and that bFGF could support the surveillance of treatment response.

## Data availability statement

The datasets presented in this study can be found in online repositories. The names of the repository/repositories and accession number(s) can be found below: https://www.ncbi.nlm.nih.gov/geo/, GSE234647.

## Ethics statement

The studies involving humans were approved by Ethics committee of the Medical University of Vienna. The studies were conducted in accordance with the local legislation and institutional requirements. The human samples used in this study were acquired from gifted from another research group. Written informed consent for participation was not required from the participants or the participants’ legal guardians/next of kin in accordance with the national legislation and institutional requirements.

## Author contributions

WM performed study concept. KB, VH and FP performed experimental analysis, interpretation of data, parts of the bioinformatic analysis and statistical analysis. DC performed RNA-seq analysis including differential expression and gene-set enrichment analysis. ER, HH and GW contributed to experiments; LB and MP provided resources and clinical expertise. KB, VH and FP wrote the manuscript. WM performed review and revision of the manuscript. WM and GO provided funding acquisition. All authors contributed to the article and approved the submitted version.
