# Supplementary material for: Synergism of the receptor tyrosine kinase Axl with ErbB receptors mediates resistance to regorafenib in hepatocellular carcinoma
Source: Front Oncol. 2023 Sep 8;13:1238883. doi: 10.3389/fonc.2023.1238883 (PMC10514905; doi:10.3389/fonc.2023.1238883)
Supplement: Supplementary file 1 [file Table_1.docx]

Supplementary Material

**Supplementary Table 1.** Baseline patient characteristics. * some patients have >1 underlying etiologies.

| *Patient characteristics* | | **Study cohort,**  **n= 20** |
| --- | --- | --- |
| Sex, n (%) | | |
|  | Male | 18 (90%) |
|  | Female | 2 (10%) |
| Aetiology *, n (%) | | |
|  | Viral | 8 (40%) |
|  | NAFLD | 5 (25%) |
|  | ARLD | 11 (55%) |
|  | Other | 1 (5%) |
| BMI, kg*m^-2^, mean ± SD | | 26.76 ± 3.41 |
| Prior surgery/ablative therapy, n (%) | | 14 (70%) |
| Cirrhosis, n (%) | | 16 (80%) |
| CTP score, points, mean ± SD | | 6.95 ± 1.56 |
|  | A, n (%) | 7 (35%) |
|  | B, n (%) | 11 (55%) |
|  | C, n (%) | 2 (10%) |
| Vascular invasion, n (%) | | 5 (25%) |
| Extrahepatic spread, n (%) | | 8 (40%) |
| ECOG PS, n (%) | | |
|  | 0 | 15 (75%) |
|  | 1 | 5 (25%) |
|  | 2 | 0 (0%) |
| BCLC stages, n (%) | | |
|  | A | 0 (0%) |
|  | B | 2 (10%) |
|  | C | 18 (90%) |
|  | D | 0 (0%) |
| Type of TKI | | |
|  | Sorafenib | 11 Patients |
|  | Regorafenib | 9 Patients |
| Laboratory parameters, median (IQR) | | |
|  | CRP, mg/dL | 1.005 (1.9825) |
|  | AFP, ng/mL | 36.05 (2141.15) |

*Abbreviations: AFP alpha fetoprotein; ARLD alcohol-related liver disease; BCLC Barcelona Clinic Liver Cancer; BMI body mass index; CRP C-reactive protein; CTP Child-Turcotte-Pugh score; ECOG PS Eastern Cooperative Oncology Group Performance Status; Ig immunoglobulin; IQR interquartile range; NAFLD non-alcoholic fatty liver disease; SD standard deviation*
