# Supplementary material for: Synergism of the receptor tyrosine kinase Axl with ErbB receptors mediates resistance to regorafenib in hepatocellular carcinoma
Source: Front Oncol. 2023 Sep 8;13:1238883. doi: 10.3389/fonc.2023.1238883 (PMC10514905; doi:10.3389/fonc.2023.1238883)
Supplement: Supplementary file 2 [file Presentation_1.pptx]

## Slide 1
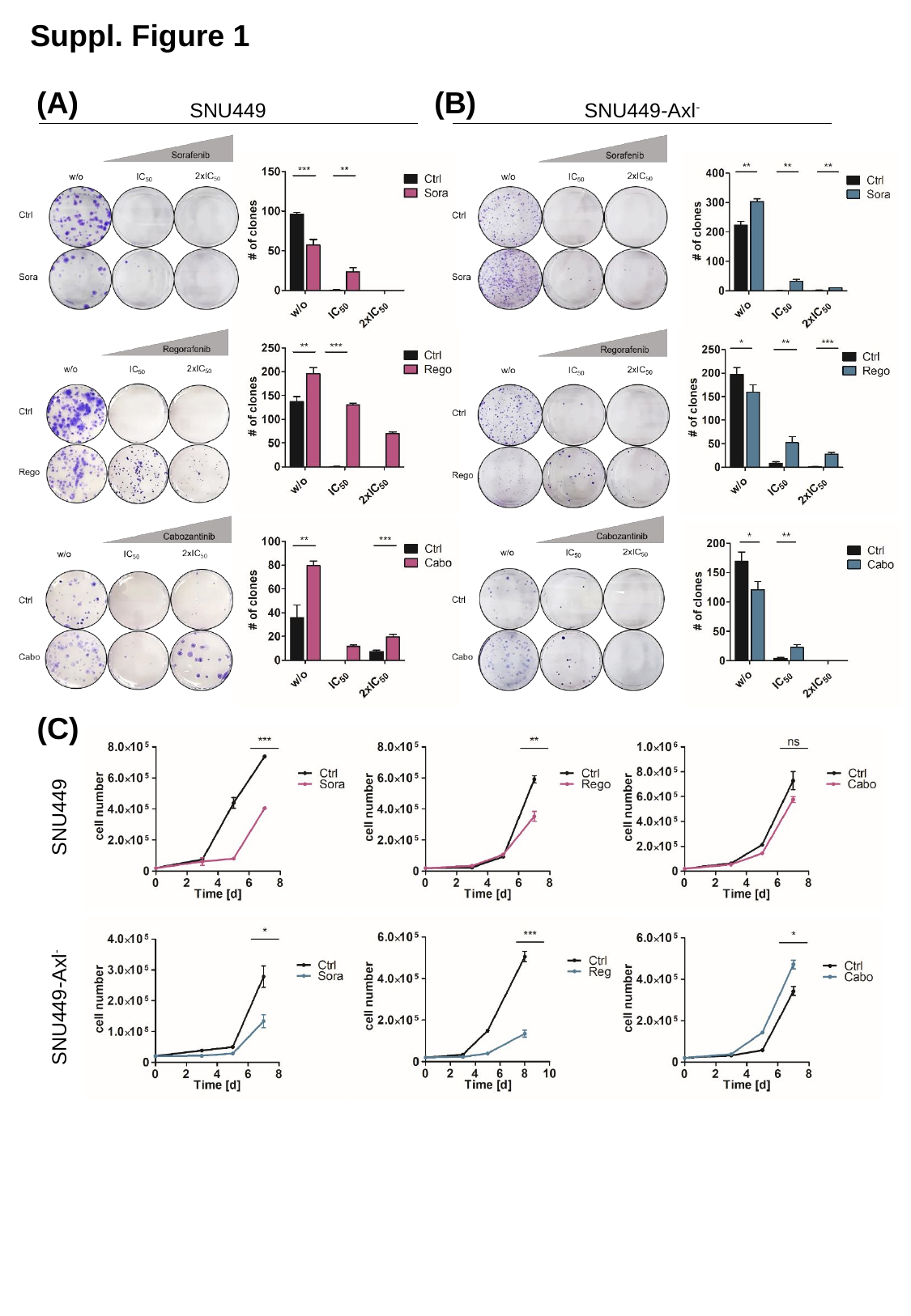

Suppl. Figure 1
(A)
(B)
SNU449
SNU449-Axl-
(C)
SNU449
SNU449-Axl-

## Slide 2
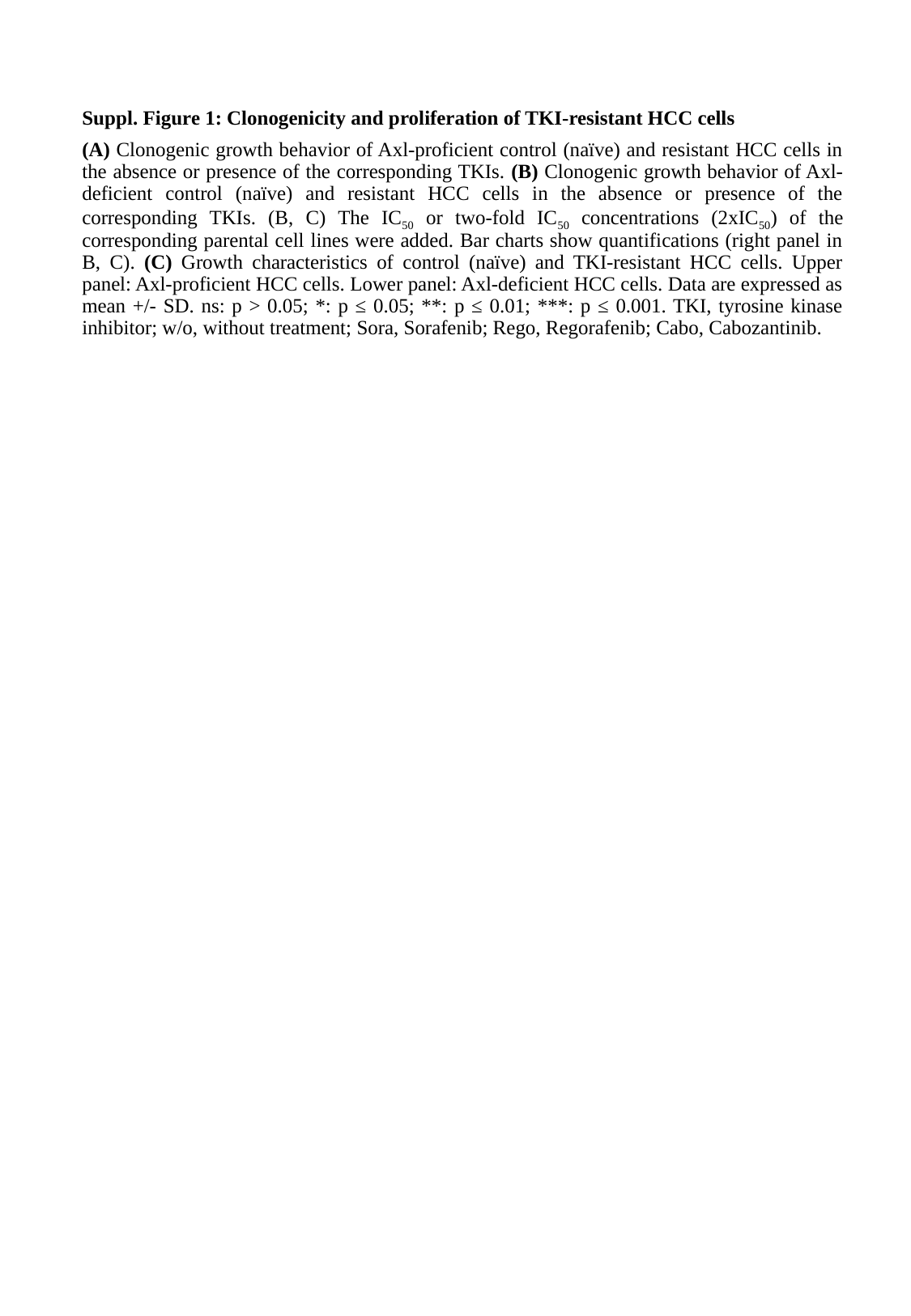

Suppl. Figure 1: Clonogenicity and proliferation of TKI-resistant HCC cells
(A) Clonogenic growth behavior of Axl-proficient control (naïve) and resistant HCC cells in the absence or presence of the corresponding TKIs. (B) Clonogenic growth behavior of Axl-deficient control (naïve) and resistant HCC cells in the absence or presence of the corresponding TKIs. (B, C) The IC50 or two-fold IC50 concentrations (2xIC50) of the corresponding parental cell lines were added. Bar charts show quantifications (right panel in B, C). (C) Growth characteristics of control (naïve) and TKI-resistant HCC cells. Upper panel: Axl-proficient HCC cells. Lower panel: Axl-deficient HCC cells. Data are expressed as mean +/- SD. ns: p > 0.05; *: p ≤ 0.05; **: p ≤ 0.01; ***: p ≤ 0.001. TKI, tyrosine kinase inhibitor; w/o, without treatment; Sora, Sorafenib; Rego, Regorafenib; Cabo, Cabozantinib.

## Slide 3
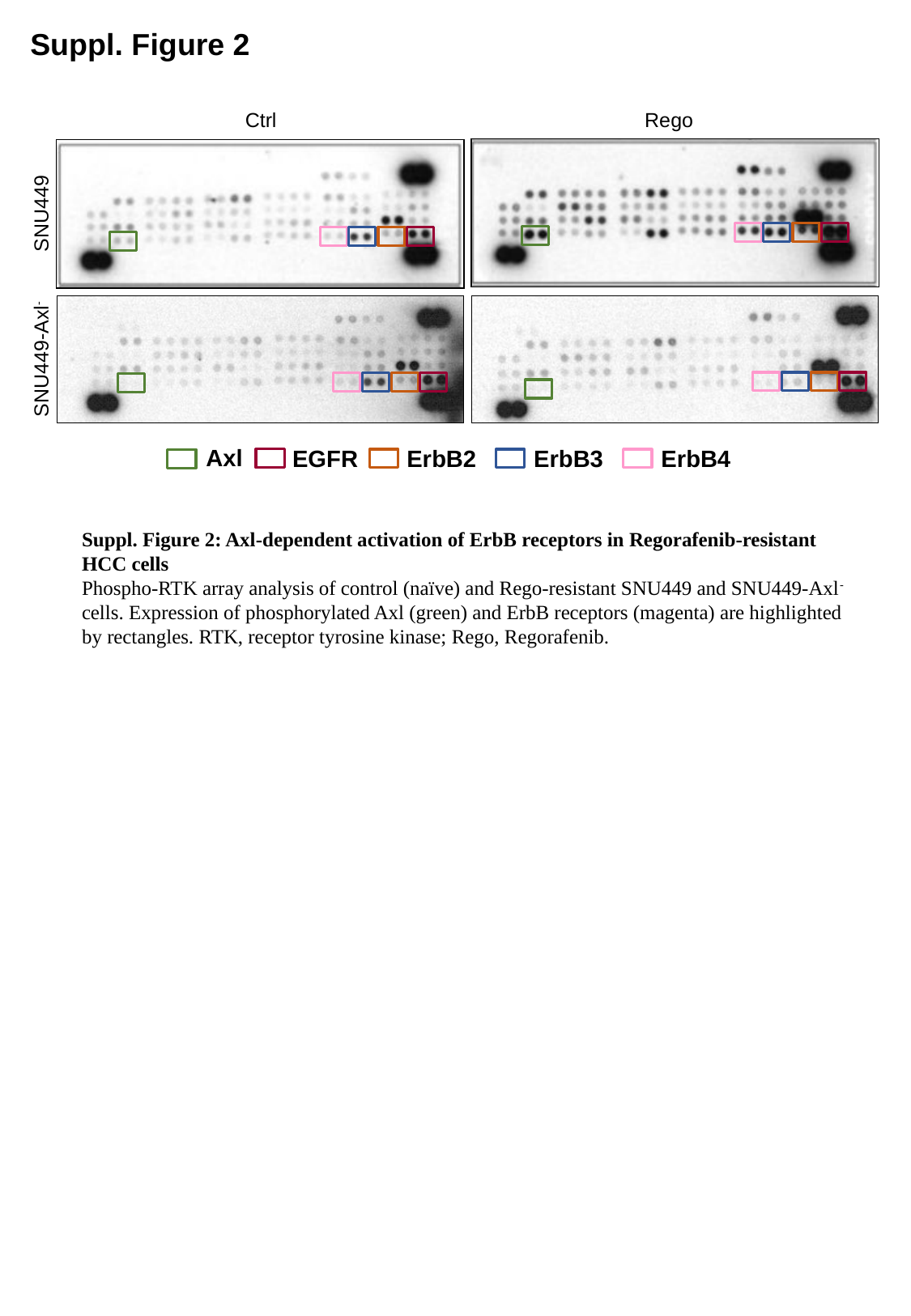

Suppl. Figure 2
Ctrl
Rego
SNU449
SNU449-Axl-
Axl
EGFR
ErbB2
ErbB3
ErbB4
Suppl. Figure 2: Axl-dependent activation of ErbB receptors in Regorafenib-resistant HCC cells
Phospho-RTK array analysis of control (naïve) and Rego-resistant SNU449 and SNU449-Axl- cells. Expression of phosphorylated Axl (green) and ErbB receptors (magenta) are highlighted by rectangles. RTK, receptor tyrosine kinase; Rego, Regorafenib.

## Slide 4
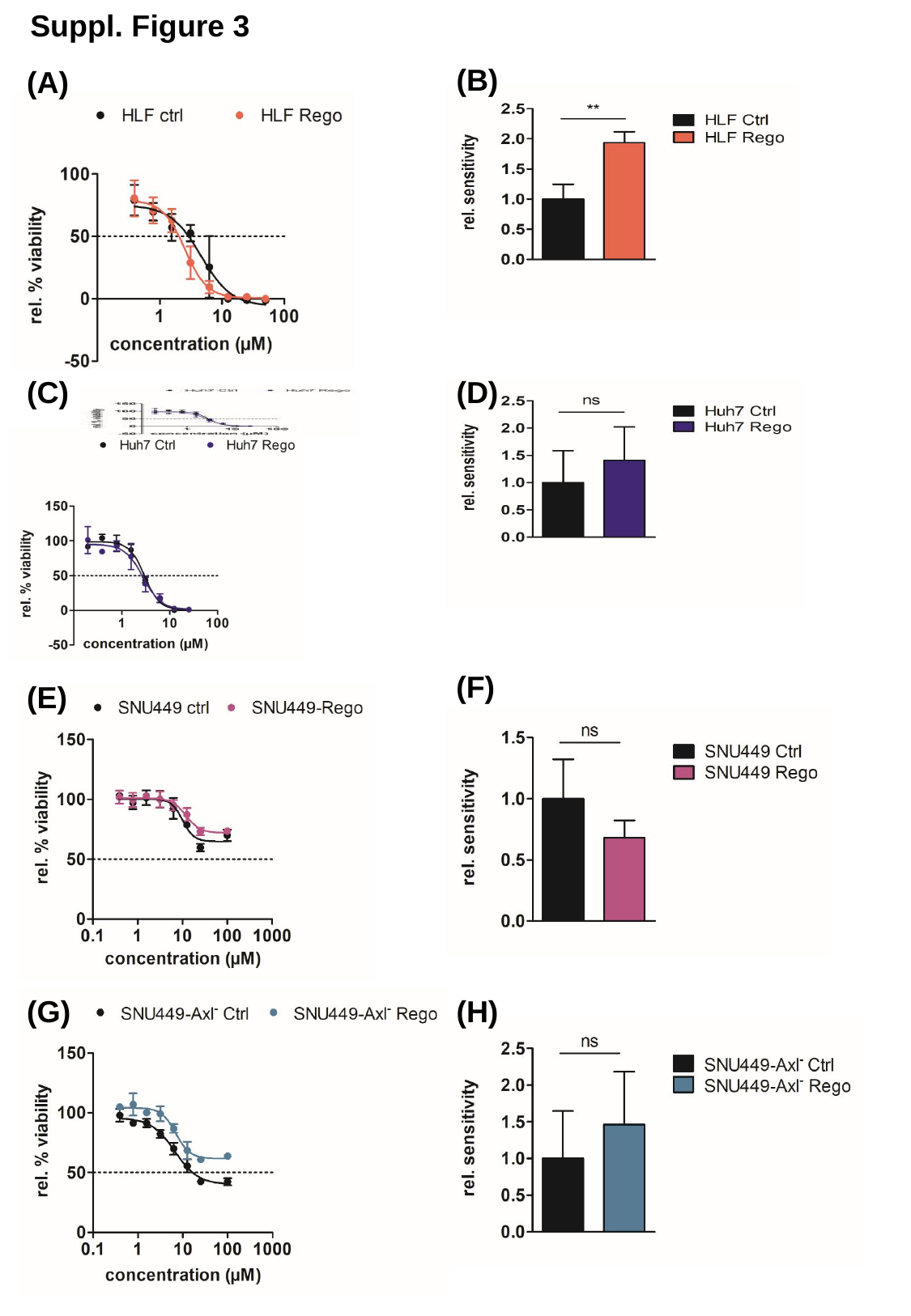

Suppl. Figure 3
(B)
(A)
(C)
(D)
(F)
(E)
(G)
(H)

## Slide 5
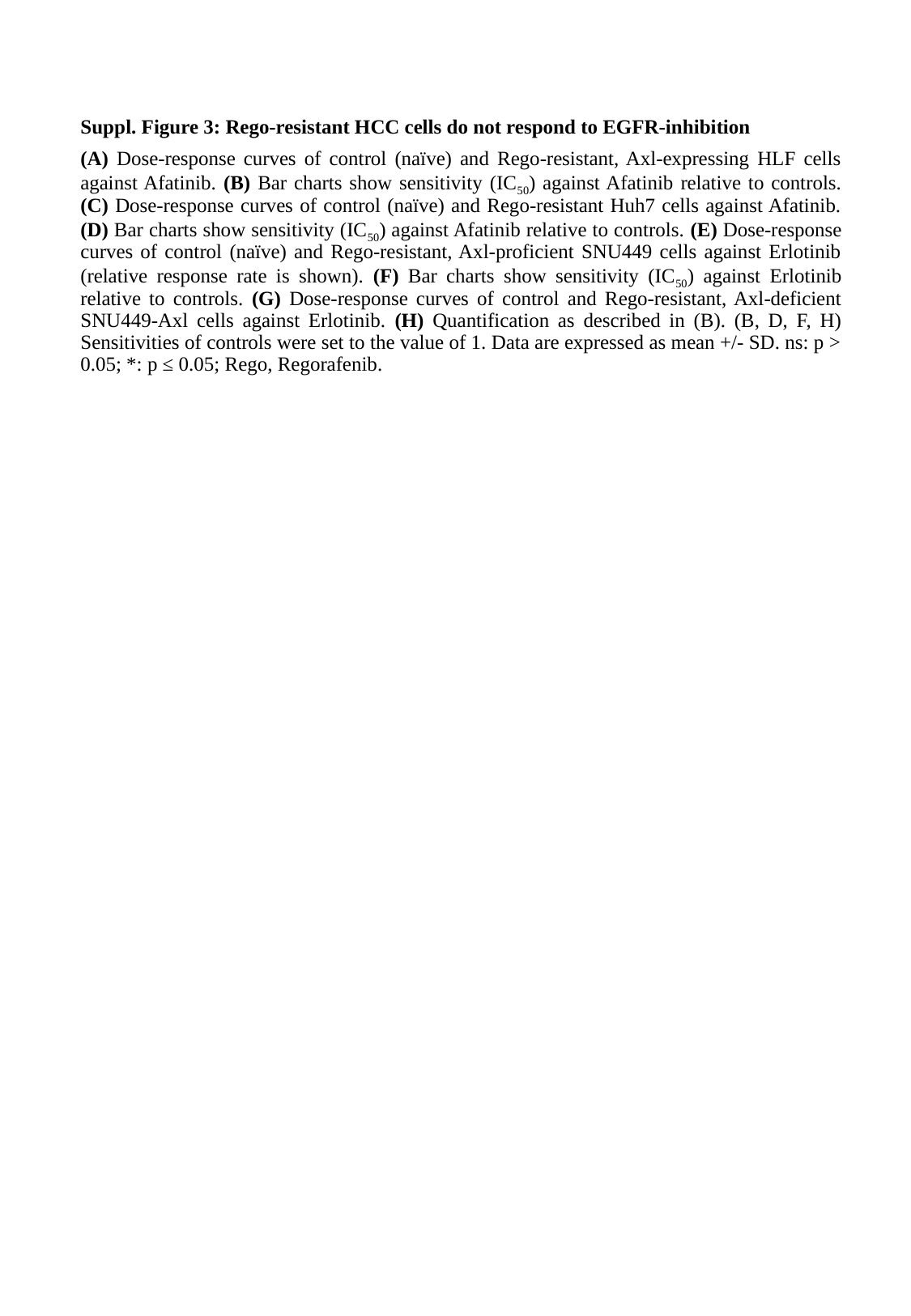

Suppl. Figure 3: Rego-resistant HCC cells do not respond to EGFR-inhibition
(A) Dose-response curves of control (naïve) and Rego-resistant, Axl-expressing HLF cells against Afatinib. (B) Bar charts show sensitivity (IC50) against Afatinib relative to controls. (C) Dose-response curves of control (naïve) and Rego-resistant Huh7 cells against Afatinib. (D) Bar charts show sensitivity (IC50) against Afatinib relative to controls. (E) Dose-response curves of control (naïve) and Rego-resistant, Axl-proficient SNU449 cells against Erlotinib (relative response rate is shown). (F) Bar charts show sensitivity (IC50) against Erlotinib relative to controls. (G) Dose-response curves of control and Rego-resistant, Axl-deficient SNU449-Axl cells against Erlotinib. (H) Quantification as described in (B). (B, D, F, H) Sensitivities of controls were set to the value of 1. Data are expressed as mean +/- SD. ns: p > 0.05; *: p ≤ 0.05; Rego, Regorafenib.

## Slide 6
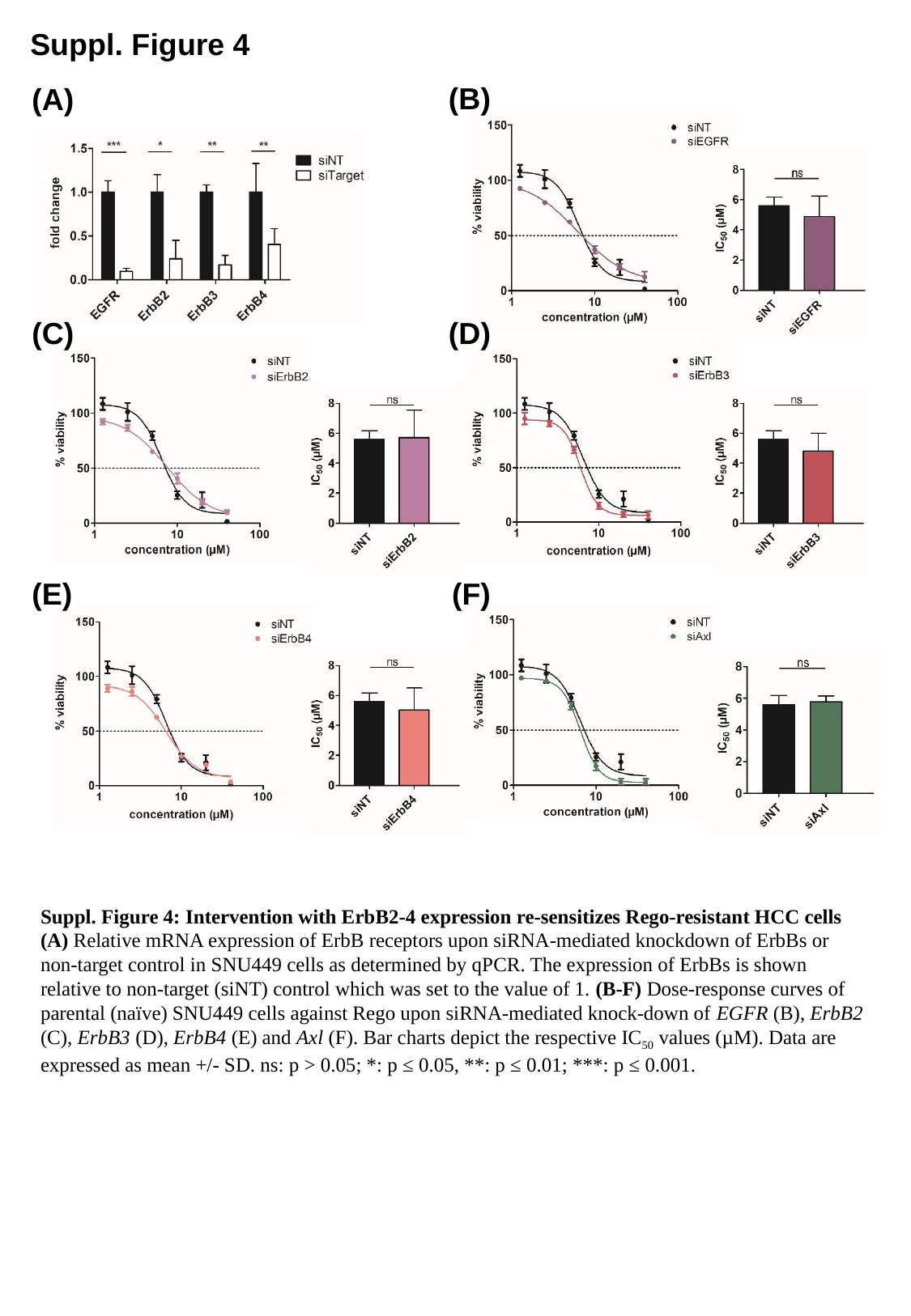

Suppl. Figure 4
(B)
(A)
(C)
(D)
(E)
(F)
Suppl. Figure 4: Intervention with ErbB2-4 expression re-sensitizes Rego-resistant HCC cells
(A) Relative mRNA expression of ErbB receptors upon siRNA-mediated knockdown of ErbBs or non-target control in SNU449 cells as determined by qPCR. The expression of ErbBs is shown relative to non-target (siNT) control which was set to the value of 1. (B-F) Dose-response curves of parental (naïve) SNU449 cells against Rego upon siRNA-mediated knock-down of EGFR (B), ErbB2 (C), ErbB3 (D), ErbB4 (E) and Axl (F). Bar charts depict the respective IC50 values (µM). Data are expressed as mean +/- SD. ns: p > 0.05; *: p ≤ 0.05, **: p ≤ 0.01; ***: p ≤ 0.001.

## Slide 7
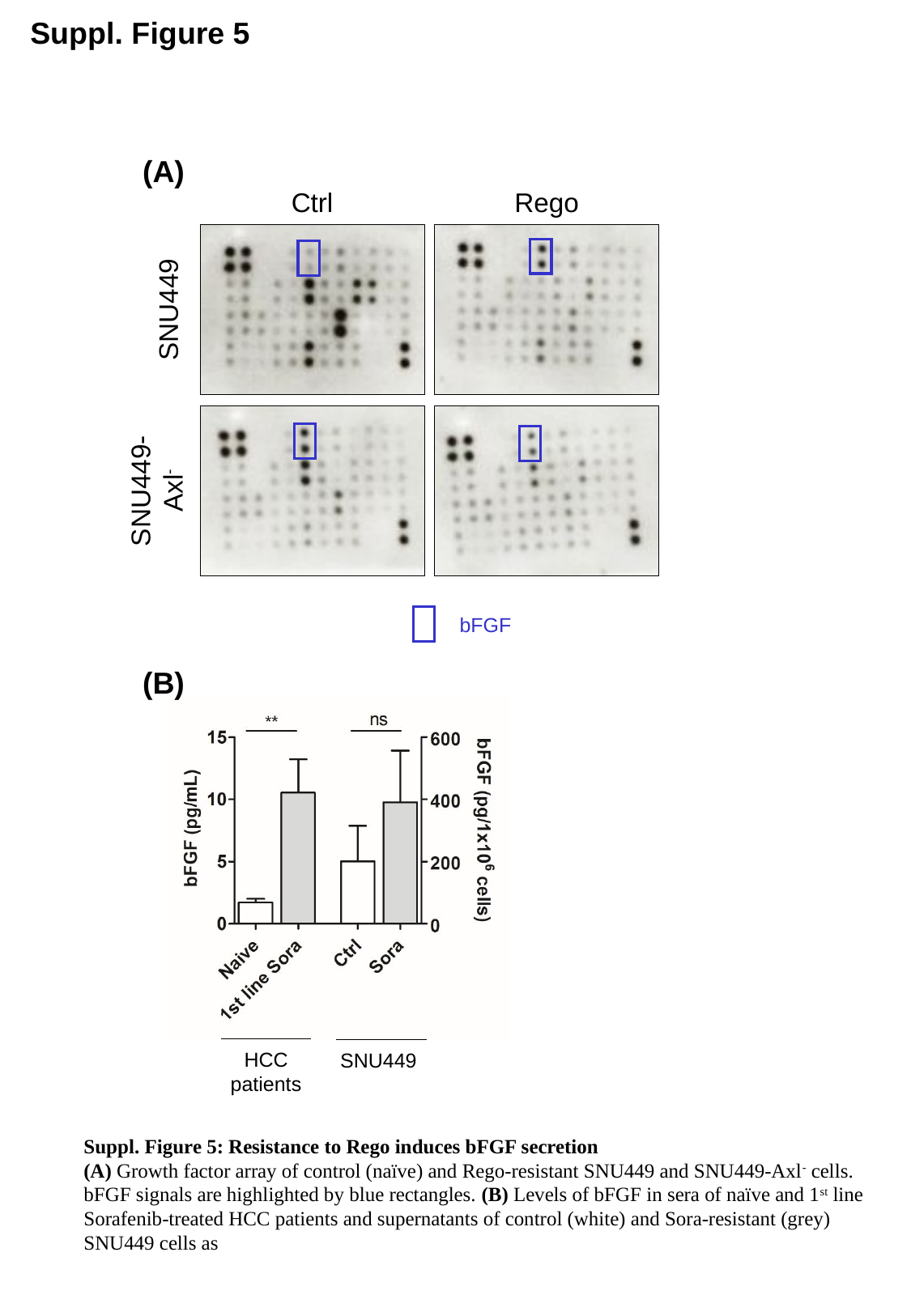

Suppl. Figure 5
(A)
Ctrl
Rego
SNU449
SNU449-Axl-
bFGF
(B)
HCC patients
SNU449
Suppl. Figure 5: Resistance to Rego induces bFGF secretion
(A) Growth factor array of control (naïve) and Rego-resistant SNU449 and SNU449-Axl- cells. bFGF signals are highlighted by blue rectangles. (B) Levels of bFGF in sera of naïve and 1st line Sorafenib-treated HCC patients and supernatants of control (white) and Sora-resistant (grey) SNU449 cells as
